# Pre-clinical Mouse Models of Neurodegenerative Lysosomal Storage Diseases

**DOI:** 10.3389/fmolb.2020.00057

**Published:** 2020-04-15

**Authors:** Jacob M. Favret, Nadav I. Weinstock, M. Laura Feltri, Daesung Shin

**Affiliations:** Hunter James Kelly Research Institute, Department of Biochemistry and Neurology, Jacobs School of Medicine and Biomedical Sciences, University at Buffalo, Buffalo, NY, United States

**Keywords:** lysosomal diseases, preclinical mouse models, HSCT, enzyme replacement therapy, gene therapy, chaperone therapy, substrate reduction therapy

## Abstract

There are over 50 lysosomal hydrolase deficiencies, many of which cause neurodegeneration, cognitive decline and death. In recent years, a number of broad innovative therapies have been proposed and investigated for lysosomal storage diseases (LSDs), such as enzyme replacement, substrate reduction, pharmacologic chaperones, stem cell transplantation, and various forms of gene therapy. Murine models that accurately reflect the phenotypes observed in human LSDs are critical for the development, assessment and implementation of novel translational therapies. The goal of this review is to summarize the neurodegenerative murine LSD models available that recapitulate human disease, and the pre-clinical studies previously conducted. We also describe some limitations and difficulties in working with mouse models of neurodegenerative LSDs.

## Introduction

The lysosome orchestrates a number of cellular homeostatic processes, primarily focused on the catabolism of diverse macromolecules. Lysosomes contain more than 50 unique acid hydrolases, each facilitating the degradation of specific metabolites including glycosides, sulfates, phosphates, various lipids, phospholipids, proteins and nucleic acids. Lysosomal hydrolases are synthesized in the ER, tagged with a mannose-6-phosphate (M6P) residue in the Golgi apparatus, and properly trafficked to the lysosome via M6P receptors. These receptors are also expressed at the plasma membrane of cells, thus allowing for the internalization of secreted lysosomal enzymes from the environment (Platt and Walkley, 2004).

Lysosomal storage diseases (LSDs) are a heterogeneous group of inherited diseases, caused by mutations leading to a deficiency of lysosomal hydrolases or transporters. LSDs lead to the accumulation of specific substrates within the lysosomal compartment, consequentially triggering a number of secondary cellular responses that result in cellular dysfunction, death and tissue damage. Substrate storage causes a wide range of perturbed cellular functions including the loss-of-function in housekeeping processes and pathways, modulation of signal transduction cascades, aberrant activation of inflammatory responses, impaired intracellular trafficking of vesicles and membrane-bound proteins, and disequilibrium of autophagic flux (Walkley, 2009). Although the nature of substrate accumulation and generalized lysosomal dysfunction is seemingly intuitive, there are a number of intriguing unanswered questions in the field of LSDs. For example, though lysosomal hydrolases are generally ubiquitously expressed, the lysosomal storage often varies even among neighboring cells (Marques and Saftig, 2019). This may be explained by a number of factors including the ability of cells to upregulate the lysosomal-autophagy pathway (Schultz et al., 2011) or whether cells have alternate strategies to dispose of stored material (Schultz et al., 2011; Ferraz et al., 2016).

Due to the heterogeneous accumulation of enzymatic substrates among multiple tissues and organs, the phenotypes among LSDs vary widely and often include visceral, ocular, hematologic, skeletal and neurological manifestations. In particular, those related to the involvement of the central nervous system (CNS) may cause progressive neurodegeneration and severe cognitive impairment. Approximately two thirds of LSD patients display CNS imparment to some extent, resulting in progressive neurodegeneration (Parenti et al., 2013). Post-mitotic cells such as neurons rely heavily on the endolysosomal and autophagic systems to prevent accumulation of debris that would otherwise become toxic. Furthermore, the extreme anatomical architecture of neurons makes the actual sequestration and degradation of substrates very challenging, as lysosomes have to travel very long distances from the cell soma to distal axons and dendrites. On top of all of this, lysosomal and autophagic functions decrease in aging (Cuervo and Dice, 2000; Lynch and Bi, 2003; Kurz et al., 2008), further pressuring the system’s efficiency. Therefore, it is not surprising that many LSDs have nervous system involvement and that various aging-related neurodegenerative diseases are caused, at least in part, by endolysosomal dysfunction. These include Parkinson’s (Anglade et al., 1997), Alzheimer’s (Nixon et al., 2005), Huntington’s diseases (Rudnicki et al., 2008), and amyotrophic lateral sclerosis (Sasaki, 2011) caused by the accumulation of aberrant or misfolded proteins. Due to the common underlying mechanisms between LSDs and neurodegenerative diseases, the development of novel treatments for LSDs may have supplemental benefits for a larger spectrum of neurodegenerative conditions. Here, we review currently available mouse models for neurodegenerative LSDs and discuss how those models have been used for pre-clinical trials and have helped move therapies forward.

## Main Text

### Therapeutic Approaches for LSDs

**FIGURE 1:**
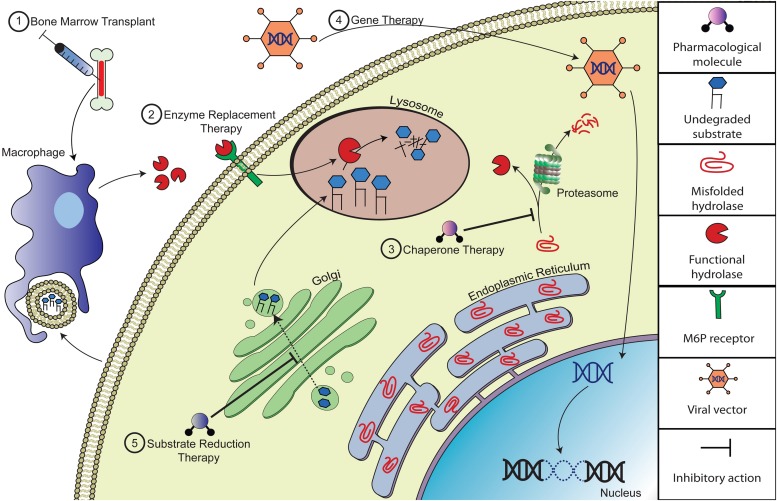
Depiction of potential therapeutic approaches to correct substrate accumulation of lysosomal storage diseases. (1) Bone marrow transplantation (BMT) alleviates storage by introducing normal donor-derived macrophages that will cross-correct with viable lysosomal hydrolases and/or phagocytize excess substrate. (2) Enzyme replacement therapy (ERT) compensates for the loss of endogenous hydrolase activity by providing recombinant enzyme, which could be taken up by mannose/M6P receptors on the cell surface. (3) Pharmacologic chaperone therapy (PCT) can improve the catalytic activity of misfolded lysosomal enzyme by promoting folding and acquisition of functional conformation of the nascent mutant peptide and thus evading pre-mature degradation. (4) Gene therapy (GT) is a therapeutic approach designed to deliver recombinant DNA to enzyme deficient cells, often via viral vector therapy. (5) Substrate reduction therapy (SRT) involves delivery of small molecule inhibitors that reduce biosynthesis of the specific accumulating substrate.

During the past three decades, research in the field of LSDs has made marked progress. Innovation of novel therapeutic approaches has given hope to many where historically the outlook has been bleak. Some LSDs are now treatable, though most cannot be treated after symptoms begin. The major strategy implemented in the treatment of LSDs is to restore or replace the defective enzyme’s activity. These modalities include hematopoietic stem cell transplantation (HSCT), enzyme replacement therapy (ERT), pharmacological chaperone therapy (PCT) and gene therapy (GT). Alternative approaches include substrate reduction therapy (SRT), based on reducing the synthesis of the substrates stored in the lysosomes (Figure 1; Eisenstein, 2016).

#### Hematopoietic Stem Cell Transplantation Therapy

Hematopoietic stem cell therapy (HSCT) is one of the more common treatments of LSDs. HSCT is the main therapy for mucopolysaccharidosis (MPS) I and Krabbe disease, and has been used in other LSDs including MPS II, MPS IVA, MPS VII, metachromatic leukodystrophy and fucosidosis (Poswar et al., 2019). HSCT allows for the delivery and engraftment of donor derived stem cells in patients with LSDs. The healthy cells repopulate in specific tissues and secrete functional lysosomal hydrolases into the extracellular space and into the blood circulation. The secreted normal enzyme may be taken up by the endogenous cells to cross-correct the enzyme deficiency of the mutated cells (Prasad and Kurtzberg, 2009). Additional benefits of HSCT have also been speculated to be immunomodulatory. HSCT-derived macrophages, which have functional lysosomal enzymes, may be able to better phagocytize dying cells/debris. Generally, earlier HSCT leads to improved outcomes but is only efficacious if delivered to pre-symptomatic patients, presumably before the occurrence of irreversible cell damage. This is particularly true for the neurologic symptoms of LSDs. Recently, genetically modified HSCT, termed HSCGT (Hematopoietic Stem Cell - Gene Therapy) has been used successfully to treat metachromatic leukodystrophy patients (MLD) (Biffi et al., 2013; Lorioli and Biffi, 2015) who re-introduced the patients’ own CD34+cells with lentivirus-transfected cells, overexpressing arylsulfatase A (ARSA). Similarly, MPS IIIA phase I/II clinical trials of *SGSH* are currently underway based on an HSCGT pre-clinical study in MPSIIIA mice (Bhaumik et al., 1999; Ellison et al., 2019). Alternatively, gene editing approaches have also been implemented to modify HSCT or induced pluripotent stem cells (iPSCs) (Mandal et al., 2014; Christensen and Choy, 2017). Other non-LSDs like cerebral adrenoleukodystrophy (Eichler et al., 2017), have had success treating patients with HSCGT which has garnered significant attention and excitement. Furthermore, alternative stem cell transplantation, like oligodendrocyte progenitor cell implantation (Scaravilli and Jacobs, 1981, 1982; Scaravilli and Suzuki, 1983; Givogri et al., 2006) has also been explored in MLD.

#### Enzyme Replacement Therapy

Enzyme replacement therapy (ERT) consists of periodic intravenous infusions of recombinant lysosomal enzyme in patients with LSDs. The first use of ERT was the use of glucocerebrosidase for Gaucher disease in 1991 (Barton et al., 1991). ERT has since been used for Farber’s disease, Pompe disease, MPS types I, II, IVA, VI, and VII and lysosomal acid lipase deficiency, and is currently being developed for others (Poswar et al., 2019). It is now possible to mass produce purified enzyme due to advances in recombinant DNA techniques. Once injected, the normal recombinant enzymes are distributed to tissues, internalized by endocytosis and targeted to the lysosomal compartment, where they replace the defective enzyme. Receptor-mediated endocytosis underlies the cellular uptake of lysosomal enzymes with mannose residues that bind to mannose receptors on the cell surface, as well as M6P residues that bind to M6P receptors (Platt et al., 2018). A major limitation of ERT is that not all organs are freely accessible to the administered enzyme. Recombinant enzymes are large molecules that do not passively diffuse across membranes. Consequently, the enzyme is unable to reach therapeutic concentrations in some of the key target tissues. Most notably, there is poor efficacy of recombinant enzyme in reaching the CNS, due in part to restricted diffusion across the blood brain barrier (BBB). To overcome the challenges presented by the BBB, it has been tried to make modified enzymes to be transported through existing systems, such as the insulin or transferrin receptors. Moreover, direct administration of recombinant enzyme into the CNS has proven an effective method of distribution (Platt et al., 2018; Kohlschütter et al., 2019). Another major limitation of ERT is that the exogenous recombinant enzyme can elicit an immune reaction. These responses include hypersensitivity reactions, neutralizing antibodies to the recombinant enzyme and altered enzyme turnover and uptake (Brooks, 1999).

#### Pharmacological Chaperone Therapy

Pharmacological chaperone therapy (PCT) attempts to rescue reduced or absent function of mutant lysosomal protein which is misfolded or mis-trafficked. The approach uses small molecule ligands which bind and stabilize mutant enzyme. The binding of chaperone to mutant enzyme facilitates increased cellular enzyme concentrations, improved enzyme trafficking and increased lysosomal activity (Parenti et al., 2015). For example, sub-inhibitory concentrations of active site inhibitors can stabilize the mutant enzyme, which extends half-life (Platt et al., 2018). Competitive enzyme inhibitors are expected to be effective as active site specific chaperones, because of their high affinity to the catalytic domain (Fan, 2008). As a result, the enzymatic activity of the mutant protein is partially rescued. Minor increases in enzymatic activity have a favorable impact on the clearance of storage material and thus patient status and rate of disease progression. PCTs have several advantages, as compared to other therapies, as they can be administered orally, allowing for a non-invasive treatment and are non-immunogenic. Furthermore, pharmaceutical chaperones are generally small enough to diffuse passively across cell membranes and reach therapeutic concentrations in different tissues and systems, including the CNS. A major limitation of PCT is that it cannot be used for stop-codon mutations because they result in premature termination or nonsense mRNA decay. For this type of nonsense mutant, other type of small molecules that can override or read-through the stop-codons are now under development (Platt et al., 2018). The development of nonsense mutation LSD mouse models has better allows researchers to test these nonsense-suppression therapies (NST). This is of importance as many LSDs harbor nonsensense mutations. For example, greater than 50% of *CLN1* disease is caused by nonsense mutations (Miller et al., 2015).

#### Gene Therapy

Gene therapy (GT) for LSDs is a rapidly advancing field of treatment. The major approach in GT is the direct transfer of the defective gene into the cells of the patient. The normal gene product is generally delivered to the patient via a viral vector (Ohashi, 2019). The first GT for LSDs was for Gaucher disease in 1998, which resulted in transient enzymatic expression in the patient (Dunbar et al., 1998). However, retroviruses generally only infect mitotically active cell types. This property seriously limits the usefulness of retroviruses as the vector of GT for CNS disease, in which most cells are post-mitotic. To overcome this limitation, other viral vectors have been studied and implemented, including adeno-associated virus (AAV) and lentivirus (LV) (Suzuki, 2004). AAV has been especially effective in correcting genetic diseases, quickly becoming one of the most promising viral vectors for the treatment of LSDs. AAVs are capable of infecting cells that are not going through mitosis and persist primarily as non-integrative episomal units. Therefore, various AAV serotypes have been developed with particular tropism for cells and tissues of interest, including neurons and glia, and tested in pre-clinical mouse models of LSDs (Bailey et al., 2018). AAV-mediated GT successfully improved the phenotypes of GM1 gangliosidosis, MPS I & IIIB, Sandhoff disease, metachromatic leukodystrophy, and Krabbe disease (Gonzalez and Baldo, 2017). Based on the positive results from these animal models, multiple phase I/II clinical trials are currently being conducted, many of which have promising results. Of note, multiple technical approaches exist regarding the site of injection, particularly in regards to treating the CNS in neurodegenerative LSDs. Some approaches have implemented systemic intravenous GT administration (Fu et al., 2016), while others attempted more localized intracerebral (Winner et al., 2016) or intrathecal administration of the viral vector (Karumuthil-Melethil et al., 2016; Bey et al., 2017), or an approach across multiple sites (Marshall et al., 2018). Furthermore, major advances in the realm of GT in recent years has been centered on the bourgeoning field of gene editing via CRISPR/Cas9 and zinc finger nuclease (ZFN) technologies. These approaches either introduce exogenous genetic material or repair the defunct endogenous locus. Alternatively, non-viral methods of introducing the gene of interest such as electro gene therapy, the phiC31, minicircle and Sleeping Beauty gene transfer systems have been implemented pre-clinically to test the efficacy of non-viral gene therapy (Aronovich et al., 2007, 2009; Osborn et al., 2008; Stilhano et al., 2015). General advantages of GT over other approaches are the stable and long-term production of therapeutic protein. GT therefore has advantages compared to ERT and small molecule therapies, which require life-long treatment. Concerns of GT do remain, including the risk that the modification of genomic DNA in the patient increases the risk of carcinogenesis (Chandler et al., 2017a). Furthermore, the expression of supra-physiologic levels of enzyme may cause unintended side effects. Finally, one challenge remaining in certain cases is the broad distribution of the viral vector to all the tissues involved (Cearley and Wolfe, 2007).

#### Substrate Reduction Therapy

Substrate reduction therapy (SRT) often uses small-molecule inhibitors to partially inhibit specific steps of the biosynthetic pathways of substrates that accumulate in LSDs (Radin, 1996). Two SRT drugs for Gaucher disease (miglustat and eliglustat tartrate) have been approved and others are undergoing clinical trials (Coutinho et al., 2016). Since SRT drugs are orally administered, SRT does not involve an invasive delivery. Due to their general low molecular mass, SRT drugs are non-immunogenic, and mostly can cross the BBB. Although in principle SRT could be useful for all LSDs, it is restricted to the one whose specific upstream biosynthetic pathways have been identified. An intriguing expansion of this approach can be thought of as genetic SRT, in which small interfering RNAs could theoretically be used to silence enzymes responsible for the production of LSD accumulated substrates (Coutinho et al., 2016). Other forms of SRT include dietary modification and restriction of substrate intake (Denny et al., 2010; Soga et al., 2015).

#### Combination Therapy

Since LSDs are multisystem disorders, multiple therapies may be required to effectively treat different components of disease pathology. Due to the partial effect of different modalities of therapies, combining different forms of therapy can be more effective than a single approach. Therefore, combination therapies to address the diverse symptoms of LSDs may be required (Platt et al., 2018). In animal models, the efficacy of combination therapy is actively being investigated, and in some cases the synergy that is reported is striking. For example, combining different forms of therapy has had profound improvements of survival on the *twitcher* mouse model of Krabbe disease (KD). In particular, the use of HSCT has been particularly efficacious in synergizing with viral-directed gene therapy. The combination of HSCT and GT has been replicated by many different labs with different viral vectors and regiments of delivery (Hawkins-Salsbury et al., 2015; Rafi et al., 2015b).

#### Autophagy Modulators

Additional strategies for treating LSDs involve targeting common mechanisms involved in LSD and neurodegenerative pathophysiology. The benefit of these approaches, which are downstream of the specific lysosomal hydrolase mutation, is that they can theoretically be applied broadly. For example, defects in autophagy have been associated with a large number of LSDs (Settembre et al., 2008; Seranova et al., 2017). Recent attempts to increase autophagy in LSDs has been successfully employed in a variety of pre-clinical models including overexpression of the MiTF transcription factors TFEB and TFE3 (Spampanato et al., 2013; Rega et al., 2016) or by manipulation of the mTOR pathway (Bartolomeo et al., 2014). These approaches also have the added benefit of increasing lysosomal exocytosis, which is thought to decrease the lysosomal storage burden and associated pathology. Alternatively, for neurodegenerative LSDs, attempts to decrease the accumulation of misfolded proteins and restore proper autophagy have been employed (Monaco et al., 2020). While most of these trials remain in the pre-clinical stage, their clinical applications seem promising.

#### Other Therapies Under Development

A large number of alternative therapeutic strategies relating to inflammation and neurodegeneration have also been tested in various pre-clinical trials. Prolonged neuroinflammation contributes to neuronal degeneration and can exacerbate the LSD phenotype and pre-clinical trials have exhibited the beneficial effects of non-steroidal anti-inflammatory drugs (NSAIDs) and other anti-inflammatory (AI) agents (Stein et al., 2015; Dannhausen et al., 2018). Some pre-clinical trials target proteins involved in modulating reactions to symptoms of the particular LSD; for example abolishing macrophage-inflammatory protein (MIP)-1 activity (Wu and Proia, 2004) to attenuate inflammation. LSDs are also often accompanied by oxidative stress, which can be treated with pharmacologic or dietary antioxidants (Wei et al., 2011; Hawkins-Salsbury et al., 2012; Saha et al., 2012; Donida et al., 2017). Akin to other neurodegenerative diseases such as Alzheimer’s disease and Huntington’s disease, LSDs can elicit aberrant neuronal signaling leading to neurotransmitter mediated excitotoxicity. Pre-clinical trials evaluating receptor antagonism (RA), AMPA-RA (Kovacs and Pearce, 2008) and NMDA-RA (Finn et al., 2013) have improved the neurobehavioral phenotypes associated with LSDs. Furthermore, a number of pharmacological agents have been explored eliciting various effects from modulating membrane fluidity (Schultz et al., 2018), modulating Ca^2+^ (Chang et al., 2007) or cholesterol levels (Erickson et al., 2000; Pelled et al., 2003; Kim et al., 2007; Repa et al., 2007; Abi-Mosleh et al., 2009; Liu et al., 2010; Taylor et al., 2012; Hovakimyan et al., 2013; Nusca et al., 2014; Tanaka et al., 2014; Soga et al., 2015; Demais et al., 2016; Liou et al., 2016), and enhancing enzyme activity (Arroyo et al., 2014) via the use of neurosteroids (NS) (Griffin et al., 2004; Liao et al., 2009). There is also interest in facilitating sphingolipid degradation via the upregulation of heat shock proteins (HSP) (Chung et al., 2016; Kirkegaard et al., 2016).

### Mouse Models of Neurodegenerative LSDs

The majority of LSDs are associated with neurodegenerative features, that are often progressive over the course of the disease. Therapies are available only for a small subset of LSDs and have not been much effective on neurological symptoms. Mouse models have played a major role in the development and improvement of novel therapeutic modalities. Pre-clinical animal models are particularly useful as they can aid in elucidating key molecular changes involved with disease pathogenesis. Various LSD mouse models have been characterized and developed and are extensively being used for the design of novel therapeutics. The use of authentic pre-clinical animal models has been shown to be predictive of therapeutic outcomes in LSD human clinical trials, and therefore reduces the time and cost of drug development. In addition, due to the small patient numbers available when studying LSDs and other rare diseases, it is difficult to standardize endpoint measurements and generate the statistical power necessary for accurate interpretation and study design (Augustine et al., 2013). Therefore, the use of pre-clinical models is important for acquiring as much information as possible about the safety and efficacy of new therapies. Here, we categorize currently available models for neurodegenerative LSDs and elaborate on how those models have been used for pre-clinical purposes. We explore the critical role of animal models in developing novel therapies and discuss broadly the advantages and caveats of existing animal models. We restrict our focus to LSDs which include neurological disease, and specifically review the mucopolysaccharidoses (MPSs), glycoproteinoses, sphingolipidoses, lysosomal transport disorders, multiple enzyme deficiency, glycogen storage diseases, and neuronal ceroid lipofuscinosis (Table 1).

**TABLE 1:** List of LSDs having neurodegeneration and currently available pre-clinical mouse models.

Gene	Disease	Mouse model	Neurodegeneration	Recaps clinical phenotype	Pre-clinical trial use
**Mucopolysaccharidosis**					

*IDUA*	Mucopolysaccharidosis, type I	*Idua*^(–/–)^ (Clarke et al., 1997)	+	+	AAV (Hartung et al., 1999, 2004; Desmaris et al., 2004), BMT (Kuehn et al., 2015; Pievani et al., 2015), Crispr (Miki et al., 2019), ERT (Tong et al., 2017; Le et al., 2018; Ghosh et al., 2019), HSCT (Watson et al., 2014; Azario et al., 2017), LV (Di Domenico et al., 2005), NVGT (Aronovich et al., 2007, 2009; Osborn et al., 2008, 2011), RV (Chung et al., 2007)
		*Idua*^(–/–)^ (Ohmi et al., 2003)	+	+	AAV (Watson et al., 2006; Janson et al., 2014; Ou et al., 2019), BMT (Nan et al., 2012; Wolf et al., 2012), Crispr (Schuh et al., 2018), ERT (Piller Puicher et al., 2012; Pasqualim et al., 2015; Lizzi Lagranha et al., 2017), LV (Wang et al., 2009; da Silva et al., 2012; Ou et al., 2016), NVGT (Camassola et al., 2005; Stilhano et al., 2015), RV (Zheng et al., 2003; Baldo et al., 2013), ZFN (Ou et al., 2019)
		*Idua^(W392X^)* (Wang et al., 2012)	+	+	Crispr (Wang et al., 2018), ERT (Baldo et al., 2012) NST (Wang et al., 2012; Keeling et al., 2013)
		*Idua*^(–/–)^ (Mendez et al., 2015)	+	+	BT (Azario et al., 2017), HSCT (Gomez-Ospina et al., 2019)

*IDS*	Mucopolysaccharidosis type II	*Ids*^(–/–)^ (Muenzer et al., 2002)	+	+	AAV (Cardone et al., 2006; Polito and Cosma, 2009; Motas et al., 2016), ERT (Muenzer et al., 2002; Polito et al., 2010), NVGT (Friso et al., 2008), ZFN (Laoharawee et al., 2018)
		*Ids*^(–/–)^ (Jung et al., 2010)	+	+	AAV (Jung et al., 2010), ERT (Lee et al., 2011, 2014; Higuchi et al., 2012; Hong et al., 2012; Sohn et al., 2018)

*SGSH*	Mucopolysaccharidosis, type IIIA	*Mgat3*^(–/–)^ (Bhaumik et al., 1999)	+	+	AAV (Fraldi et al., 2007; Haurigot et al., 2013), AI (Arfi et al., 2011), BMT (Lau et al., 2012), LV (McIntyre et al., 2008), SRT (Roberts et al., 2010)
		*Mgat3*^(D31N)^ (Bhattacharyya et al., 2001)	+	+	AAV (Ruzo et al., 2012; Haurigot et al., 2013), ERT (Gustavsson et al., 2019), GT (Quiviger et al., 2014), SRT (Roberts et al., 2007)
		*Mgat3*^(CKO)^ (Lau et al., 2017)	+*	<*	

*NAGLU*	Mucopolysaccharidosis, type IIIB	*Naglu ^(–/–)^* (Li et al., 1999)	+	+	AAV (Cressant et al., 2004; Ribera et al., 2015), ERT (Kan et al., 2014), LV (Di Natale et al., 2005)

*HGSNAT*	Mucopolysaccharidosis, type IIIC	*Hgsnat ^(–/–)^* (Martins et al., 2015)	+	+	AAV (Tordo et al., 2018)
		*Hgsnat ^(–/–)^* (Marcó et al., 2016)	+	+	AAV (Marcó et al., 2016)

*GNS*	Mucopolysaccharidosis, type IIID	*Gns ^(–/–)^* (Roca et al., 2017)	+	+	AAV (Roca et al., 2017)

**Glycoproteinoses**					

*MAN2B1*	Alpha-Mannosidosis	*Man2b1*^(–/–)^ (Stinchi et al., 1999)	+	+	ERT (Roces et al., 2004; Blanz et al., 2008; Damme et al., 2011)

*NEU1*	Sialidosis, Type I & II	*Neu1*^(–/–)^ (de Geest et al., 2002)	+	<	
		*Neu1*^(V54M)^ (Bonten et al., 2013)	+	+	Chaperone-AAV (Bonten et al., 2013)

**Sphingolipidoses**					

*ASAH1*	Farber disease	*Asah1*^*P361R*^ (Alayoubi et al., 2013)	+	+	LV (Alayoubi et al., 2013)
		*Asah1*^(–/–)^ (Eliyahu et al., 2012)	Embryonic lethal	−	

*ASA*	Metachromatic leukodystrophy	*Asa ^(–/–)^* (Hess et al., 1996)	+	<	AI (Stein et al., 2015), ERT (Matzner et al., 2005; Matthes et al., 2012), HSCT (Biffi et al., 2004; Capotondo et al., 2012), OLP (Givogri et al., 2006), RV (Matzner et al., 2000)

*GALC*	Krabbe disease	*Galc*^*Twi*^ (Kobayashi et al., 1980)	+	+	AAV (Lin et al., 2015; Rafi et al., 2015a), AI (Luzi et al., 2009), AO (Hawkins-Salsbury et al., 2012), BMT (Luzi et al., 2005), Combination (Qin et al., 2012; Hawkins-Salsbury et al., 2015; Ricca et al., 2015), HSCT (Yeager et al., 1984, 1991; Yagi et al., 2004), Nerve Graft (Scaravilli and Jacobs, 1981, 1982; Scaravilli and Suzuki, 1983)
		*Galc*^*twi–5J*^ (Potter et al., 2013)	+	+	
		*Galc*^(H168C)^ (Luzi et al., 2001)	+	+	
		*Galc^(G270D)^* (Matthes et al., 2015)	+	+	ERT (Matthes et al., 2015)
		*Sapa^(–/–)^* (Matsuda et al., 2001)	+	+	HSCT (Yagi et al., 2005)

*GBA*	Gaucher disease, type II	*Gba*^(–/–)^ (Tybulewicz et al., 1992)	-	-	
		*Gba*^(L444P)^ (Liu et al., 1998)	-	-	
		*Gba*^(pmuts)^ (Xu et al., 2003)	+	+	
		*Gba*^(lnl/lnl)^ (Enquist et al., 2007)	+	+	Chaperone (Dasgupta et al., 2015), CM (Liou et al., 2016), ERT (Cabrera-Salazar et al., 2010), SRT (Cabrera-Salazar et al., 2012)

*GLB1*	GM1-Gangliosidosis	*Glb1^(–/–)^* (Matsuda et al., 1997)	+	+	Chaperone (Takamura et al., 2011)
		*Glb1*^(–/–)^ (Hahn et al., 1997)	+	<	BMT (Sano et al., 2005), SRT (Kasperzyk et al., 2005; Elliot-Smith et al., 2008)

*HEXA*	GM2-gangliosidosis, type I	*HexA*^(–/–)^ (Sango et al., 1995)	-	-	
		*HexA*^(–/–)^ (Yamanaka et al., 1994)	-	-	SRT (Platt et al., 1997)
		*HexA^(–/–)^ & Neu3^(–/–)^* (Seyrantepe et al., 2018)	+	+	

*HEXB*	GM2-gangliosidosis, type II	*HexB^(–/–)^* (Sango et al., 1995)	+	+	AAV (Sargeant et al., 2011; Cachon-Gonzalez et al., 2014), BMT (Norflus et al., 1998; Wada et al., 2000), CM (Pelled et al., 2003), Diet (Denny et al., 2010), HSP (Kirkegaard et al., 2016), MIP-1 (Wu and Proia, 2004), SRT (Jeyakumar et al., 1999)
		*HexB*^(–/–)^ (Sargeant et al., 2011)	+	+	

*NPC1*	Niemann-pick disease, type C1	*Npc1*^(–/–)^ (Morris et al., 1977)	+	-	AAV (Chandler et al., 2017b; Xie et al., 2017; Hughes et al., 2018), AI (Alvarez et al., 2008), AO (Fu et al., 2013), CM (Erickson et al., 2000; Repa et al., 2007; Abi-Mosleh et al., 2009; Liu et al., 2010; Taylor et al., 2012; Hovakimyan et al., 2013; Nusca et al., 2014; Tanaka et al., 2014; Soga et al., 2015; Demais et al., 2016), Diet (Jelinek et al., 2015; Soga et al., 2015), HSP (Chung et al., 2016; Kirkegaard et al., 2016), NS (Griffin et al., 2004; Liao et al., 2009), Transplant (Veyron et al., 1996)
		*Npc1*^(D1005G)^ (Maue et al., 2012)	+	+	HSP (Chung et al., 2016)
		*Npc1*^(Flox)^ (Elrick et al., 2010)	+**	<**	
		*Npc1*^(P202A & F203A)^ (Xie et al., 2011)	+	+	
		*Npc1*^(I1061T)^ (Praggastis et al., 2015)	+	<	
		*Npc1*^(1554–1004G<^*^*A*^*^)^ (Gómez-Grau et al., 2017)	+	+	

SMPD1	Niemann-pick disease, type A & B	*Asm*^(–/–)^ (Otterbach and Stoffel, 1995)	+	+	
		*Asm*^(–/–)^ (Horinouchi et al., 1995)	+	+	AAV (Passini et al., 2007), NSMA (Arroyo et al., 2014), ERT (Dodge et al., 2009)

**Lysosomal transport defects**					

*CTN5*	Cystinosis	*Ctns*^(–/–)^ (Cherqui et al., 2002)	+	<	ERT (Cherqui et al., 2002; Simpson et al., 2011), HSCT (Syres et al., 2009; Rocca et al., 2015; Gaide Chevronnay et al., 2016)

*SLC17A5*	Free sialic acid storage disorder	*Sialin*^(–/–)^ (Prolo et al., 2009)	+	+	

**Multiple enzyme deficiency**					

*SUMF1*	Multiple sulfatase deficiency	*Sumf1*^(–/–)^ (Settembre et al., 2007)	+	+	AAV (Spampanato et al., 2011)

*GNPTAB*	Mucolipidosis, type II	*Gnptab*^(–/–)^ (Ko et al., 2016)	+	+	AAV (Ko et al., 2016)
		*Gnptab^(Y888)^* (Paton et al., 2014)	+	+	
		*Gnptab^(–/–)^* (Gelfman et al., 2007)	+	<	

*MCOLN1*	Mucolipidosis, type IV	*Mcoln1*^(–/–)^ (Venugopal et al., 2007)	+	+	BMT (Walker and Montell, 2016), SRT (Boudewyn et al., 2017)

**Glycogen storage disease**					

*GAA*	Glycogen storage disease	*Gaa*^(–/–)^ (Raben et al., 1998)	+	+	AAV (Rucker et al., 2004; Zhu et al., 2005; Gatto et al., 2017; Puzzo et al., 2017), BMT (Mori et al., 2008), Chaperone (Khanna et al., 2012, 2014), ERT (Raben et al., 2001, 2002, 2010; Xu et al., 2004), SRT (Ashe et al., 2010; Douillard-Guilloux et al., 2010)
		*Gaa^(–/–)^* (Bijvoet et al., 1998)	+	+	HSCT (van Til et al., 2010)

*LAMP2*	Danon disease	Lamp2^(y/–)^*** (Tanaka et al., 2000)	+	+	

**Neuronal ceroid lipofuscinosis**					

*CLN2*	Ceroid lipofuscinosis, neuronal 2	*Cln2*^(–/–)^ (Sleat et al., 2004)	+	+	AAV (Sondhi et al., 2008; Chen et al., 2009), Anti-Apoptosis (Kim et al., 2009), ERT (Kim et al., 2008; Sleat et al., 2008; Meng et al., 2012)

*CLN3*	Ceroid lipofuscinosis, neuronal 3	*Cln3 ^(–/–)^* (Cotman et al., 2002)	+	+	AI (Dannhausen et al., 2018)
		*Cln3*^(LacZ)^ (Eliason et al., 2007)	+	+	Membrane Fluidity Modulation (Schultz et al., 2018)
		*Cln3 ^(–/–)^* (Mitchison et al., 1999)	+	+	AMPARA (Kovacs and Pearce, 2008), CaM (Chang et al., 2007)

*CLN6*	Ceroid lipofuscinosis, neuronal 6	*Cln6*^(–/–)^ (Bronson et al., 1998)	+	+	Diet (Mirza et al., 2013), LV (Jankowiak et al., 2015)

*PPT1*	Ceroid lipofuscinosis, neuronal 1	*Ppt1*^(–/–)^ (Gupta et al., 2001)	+	+	AAV (Macauley et al., 2014; Shyng et al., 2017), AO (Wei et al., 2011; Saha et al., 2012), ERT (Hu et al., 2012), NMDARA (Finn et al., 2013), SRT (Sarkar et al., 2013)
		*Ppt1*^(R151X)^ (Miller et al., 2015)	+	+	NST (Miller et al., 2015)
		*Ppt1*^(C451T)^ (Bouchelion et al., 2014)	+	+	

AAV, Adeno-associated virus; AI, Anti-Inflammatory; AMPARA, AMPA-Receptor antagonist; AO, Anti-Oxidant; BMT, Bone Marrow Transplant; BT, Blood Transfusion; CaM, Ca^*2*+^ Modulation; CM, Cholesterol Modulation; ERT, Enzyme Replacement Therapy; HSCT, Hematopoietic Stem Cell Transplant; HSP, Heat shock protein; LV, Lentivirus; NMDARA, NMDA-Receptor Antagonist; NSMA, Neutral Sphingomyelinase Activation; NS, Neurosteroids; NST, Nonsense Suppression Therapy; NVGT, Non-Viral Gene Transfer; OLP, Oligodendrocyte Progenitor Therapy; RV, Retrovirus; SRT, Substrate Reduction Therapy; ZFN, Zinc Finger Nuclease. (+) distinguishes murine models of LSD that presents neuronal degeneration or presents with analogous disease progression as seen in humans. (−) a lack of neuronal degeneration and/or comparable disease pathology. (<) Represents murine models that display a similar yet milder disease progression than human patients. *The neuronal degeneration and recaps clinical phenotype designations for the *Mgat3*^(CKO)^ model were interpreted from the conditional knockout mice model crossed to with *CMV-Cre* line; a model with ubiquitous expression across all tissue types. **The neuronal degeneration and recaps clinical phenotype designations for the *NPC1*^(Flox)^ model were interpreted from the conditional knockout mice crossed with *Pcp2-Cre* line; a driver targeting most Purkinje cells and some retinal bipolar neurons. ***The *Lamp2* gene resides on the X chromosome of mouse genome, therefore; the investigators used hemizygous null males designated as *Lamp2*^(y/–)^.

#### Mucopolysaccharidoses

The mucopolysaccharidoses (MPS) are a family of lysosomal storage diseases wherein patients have an inability to properly metabolize glycosaminoglycan’s (GAGs) resulting in toxic accumulation of undigested dermatan sulfate (DS), heparan sulfate (HS) and/or keratin sulfate (KS) in the lysosome. GAGs are complex polymers comprised of alternating sulfated or amino disaccharides attached to protein cores, and are distributed in a wide variety of tissues, including bones, cartilage and the nervous system. There are eleven enzymes involved in the degradation of GAGs and mutations in any of these enzymes can elicit one of the seven characterized MPS. MPS are classified into 7 subtypes, with varied clinical phenotypes. The incidence for all types of MPS is estimated at 1 in 20,000 live births. The degree of CNS dysfunction, if any, varies widely among the 7 subtypes, but it seems to correlate with the degree of storage of HS, a major component of the extracellular matrix of the CNS. For example, MPS III A-D (Sanfilippo A-D) manifest as primarily CNS disorders, and often present with aggressive behavior and subsequent neurologic decline. On the other end of the spectrum, MPS VI (Maroteaux-Lamy), MPS IV A-B (Morquio) and MPS IX (Natowicz syndrome) present primarily as soft tissue or skeletal disease without neurological involvement. Other MPS can have variable degrees of neurologic involvement, in addition to soft tissue and skeletal disease, including MPS I (Hurler, Hurler-Scheie, Sheie), MPS II (Hunter) and MPS VII (Sly) (Coutinho et al., 2012).

1. MPS, Type I (OMIM [Online Mendelian Inheritance in Man] #252800) is caused by mutations in *IDUA*, which encodes a glycosidase involved in degrading HS and DS. MPS I is a multisystem disorder that ranges over a continuum of severity from severe (Hurler; MPS I-H) to attenuated (Scheie; MPS I-S) symptoms. Intellectual disability and developmental delay is common in MPS I-H, and neurological involvement and learning disabilities can be present in MPS I-S (Coutinho et al., 2012). Standard of care treatment includes HSCT, which can increase survival and peripheral symptoms, and may slow the development of mild, but not of severe, cognitive impairment. ERT with laronidase (Aldurazyme) is approved for non-CNS symptoms of MPS I (Al-Sannaa et al., 2015). Two MPS I-H mouse models were generated using targeted knockout cassettes in *Idua*, as well as a knock-in nonsense mutations (*Idua*-W392X) (Clarke et al., 1997; Ohmi et al., 2003; Wang et al., 2012). Knockout MPS I mice (*Idua -/-*) exhibit cognitive and motor defects with storage of GAGs in Purkinje cells (Baldo et al., 2012). Systemic delivery of apoE-fused IDUA protein produced from erythroid/megakaryocytic cells via LV-mediated HSCGT successfully corrected metabolic and behavioral deficits in MPS I mice (El-Amouri et al., 2014). Furthermore, efforts to directly edit the genome and correct mutations are currently underway. Several groups have undertaken attempts using genome editing tools such as the CRISPR/Cas9 and ZFN systems to directly modify the endogenous *Idua* locus or introduce an exogenous *Idua* gene (Schuh et al., 2018; Wang et al., 2018; Gomez-Ospina et al., 2019). These techniques have shown preliminarily success at promoting functional IDUA and reducing GAG storage. A ZFN mediated approach of delivering *Idua* to hepatocytes under control of the albumin promoter was particularly effective in distributing functional IDUA throughout secondary tissues via cross-correction, consequently reducing storage pathology (Ou et al., 2019). The therapy is currently in phase I/II of clinical trials.
2. MPS, Type II (OMIM #309900) is caused by mutations in the X-linked gene *IDS* (iduronate 2-sulfatase), which encodes the enzyme that catabolizes DS and HS. Infants with MPS II experience neurologic symptoms in the first decade of life and often experience developmental regression after 5 years of age (Scarpa, 2018). Weekly ERT infusions of idursulfase (Elaprase) can treat somatic manifestations and improves survival but does not treat the neurological disease (Burrow and Leslie, 2008). An *Ids*^(–/–)^ mouse model of MPS II accumulate GAGs diffusely and shows neuronal necrosis in the brainstem and spinal cord by 60 weeks of age (Muenzer et al., 2002; Jung et al., 2010). AAV2/8-mediated GT restored DS activity in plasma and tissue of this null mice and cleared the accumulated GAGs in all the tissues (Motas et al., 2016). Akin to MPS I, genome editing via ZFN has been implemented as therapeutic option for MPS II, which elicited a vast improvement in neurocognition following reduction of accumulated GAGs by active re-introduction of *Ids* to hepatocytes and passive cross-correction through all tissues (Laoharawee et al., 2018). The pre-clinical success of the ZFN mediated genome editing has led to phase I/II clinical trials assessing the drugs efficacy in humans and optimal dosage as administered by intravenous injection.
3. MPS, Type III is characterized by progressive CNS degeneration and manifests clinically as developmental regression, severe intellectual disability and psychiatric manifestations. There are four subtypes of MPS III, distinguished as types ‘A-D,’ which are caused by mutations in one of four genes, all required for the proper degradation of HS (Wagner and Northrup, 2019). Unfortunately, there is no effective therapy available for any form of MPS III, aside from clinical management of neurological symptoms. Therefore, the role of animal models for the various forms of MPS III are especially important in the design of emerging and future therapies (Fedele, 2015).
  a. MPS, Type III A (OMIM # 252900) is caused by a mutation in *SGSH* (N-sulfoglucosamine sulfohydrolase), resulting in intellectual disability, seizures and hyperactivity (Wagner and Northrup, 2019). The spontaneous mutant *Mgat3*^(–/–)^ represents an authentic animal model of the disease manifesting hyperactivity and shortened lifespan akin to the human phenotype (Bhaumik et al., 1999). This animal has been used for GT and SRT studies which have successfully extended mutant lifespan and reduced associated symptomology (McIntyre et al., 2008; Roberts et al., 2010; Haurigot et al., 2013).
  b. MPS, Type III B (OMIM # 252920) is caused by a mutation in *NAGLU* (N-alpha-acetylglucosaminidase) and presents with progressive neurological deterioration and seizures (Wagner and Northrup, 2019). The KO mouse model *Naglu*^(–/–)^ had a comparatively mild phenotype (Li et al., 1999). In this knockout mouse, cellular inclusions in neurons were observed but the resulting behavioral changes were less prominent, although hypoactive behavior is apparent. This model has been used to test GT and ERT, which improved the behavior and neuropathology of *Naglu*^(–/–)^ mice (Cressant et al., 2004; Kan et al., 2014; Ribera et al., 2015).
  c. MPS, Type III C (OMIM #252930) is caused by a mutation in *HGSNAT* (Heparan acetyl-CoA:α-glucosaminide N-acetyltransferase), causing mental retardation and hyperactivity. Prominent atrophy occurs in the parieto-occipital region with a significant thinning of the corpus callosum white matter density (Wagner and Northrup, 2019). Two different *Hgsnat*^(–/–)^ mice were generated. Both of which recapitulate human disease pathology including altered locomotor capabilities, hyperactivity, decline in cognitive memory ability, and shortened lifespan (Martins et al., 2015; Marcó et al., 2016). An AAV2 variant, AAV-TT, was used in a GT study in the *Hgsnat*^(–/–)^ mouse model, and was able to correct the neurological phenotype (Tordo et al., 2018).
  d. MPS, Type III D (OMIM #252940) is caused by mutations in *GNS* (N-acetylglucosamine-6-sulfatase) resulting in hyperactivity and mental retardation. White matter lesions are particularly present in the periventricular subcortical white matter regions (Wagner and Northrup, 2019). A *Gns*^(–/–)^ mouse was generated exhibiting similar symptomology to the human disease, such as widespread neuroinflammation, reduced locomotion, and a decrease in lifespan. AAV-mediated GT of GNS to the cerebrospinal fluid in this mouse model ameliorated disease pathology, resolving lysosomal storage, neuroinflammation and behavioral phenotypes (Roca et al., 2017).

#### Glycoproteinoses

The glycoproteinoses are a group of LSDs caused by defects in the catabolism of glycoproteins containing N-linked or O-linked oligosaccharides. The degradation of glycan moieties occurs in a stepwise fashion thus the failure of one enzyme causes a complete blockade of the cycle. Therefore, most patients with glycoproteinoses present with very similar clinical findings (Michalski and Klein, 1999).

1. α-Mannosidosis (OMIM #248500) is caused by mutations in *MAN2B1* (alpha-D-mannosidase), an enzyme involved in glycoprotein metabolism that results in accumulation of undigested oligosaccharides (Beck et al., 2013). A clinical spectrum of disease exists, spanning from mild (type 1), moderate (type 2) and severe (type 3). Severe patients have primary CNS disease, primarily involving cerebellar dysfunction and ataxia, as well as severe intellectual disability and developmental regression (Michalski and Klein, 1999). ERT with recombinant human α-mannosidase (Velmanase alfa) is regarded as the standard-of-care treatment for α-mannosidosis, which has been shown to cause improvements in both biochemical and functional endpoints of disease progression (Borgwardt et al., 2018; Lund et al., 2018). *Man2b1*^(–/–)^ mice were generated, and resemble a mild form of the disease (Stinchi et al., 1999). Glycoproteins accumulate in Purkinje cells of the cerebellum as well as cortical neurons and pyramidal neurons of the hippocampus. The cerebellar pathology of *Man2b1*^(–/–)^ mice had a partial rescue in a pre-clinical trial using recombinant MAN2B1 ERT (Blanz et al., 2008; Damme et al., 2011).
2. Sialidosis, Type II (OMIM #256550) is caused by a mutation of *NEU1* (Neuraminidase-1), leading to the lysosomal accumulation of sialylated glycopeptides and oligosaccharides, manifesting in gait disturbances, corneal clouding and psychomotor retardation. Patients present with decreased cerebellum volume, as well as cortical and occipital lobe atrophy (Gultekin et al., 2018). There is no specific treatment for Sialidosis, Type II. *Neu1*^(–/–)^ and *Neu1*^(V54M)^ models of the disease were established. The phenotypes of *Neu1*^(–/–)^ mice are not similar to those found in the human disease, because there was a lack of early degeneration in cerebellar Purkinje neurons (de Geest et al., 2002). The *Neu1*^(V54M)^ models a non-neuronopathic form of the disease. Therefore, there is currently a lack of neuronopathic representative models of Sialidosis, Type II (Bonten et al., 2013).

#### Sphingolipidoses

The sphingolipidoses are characterized by the intracellular accumulation of sphingolipids. Sphingolipids are lipids that contain an aliphatic amino alcohol head group, or a structurally similar element. There are three categories of sphingolipids: ceramides, phosphosphingolipids and glycosphingolipids. These biologically active lipids form microdomains in the plasma membrane and facilitate signal transduction, cell recognition and physical protection. Accumulation of specific sphingolipids leads to the unique clinical manifestation of each sphingolipidosis. Despite their various phenotypes, all sphingolipidoses present at least some form of neuronopathic pathophysiology (Platt et al., 2018).

1. Farber lipogranulomatosis (OMIM #228000) is caused by mutations in *ASAH1* (Acid ceramidase), leads to the accumulation of lysosomal ceramide, causing neonatal joint deformities and a characteristic hoarse voice/cry, with life expectancy of approximately 2 years. Neurologic symptoms are difficult to fully characterize due to the severity of the phenotype which includes joint immobility and early death. Older patients often develop motor defects and intellectual disabilities. A more mild spectrum of disease caused by *ASAH1* mutations is categorized as spinal muscular atrophy with progressive myoclonic epilepsy, which includes progressive lower motor neuron disease in young children, accompanied by myoclonic and atonic seizures. The tissues of afflicted patients contain granulomatous and lipid-laden macrophages. The liver, spleen, lungs and heart are particularly affected with progressive CNS degeneration and impairments in psychomotor development (Bongarzone et al., 2012). There is no specific treatment for Farber disease. While a complete ‘knockout’ of ASAH1 (*Asah1-/-*) mice resulted in early embryonic lethality due to oocyte apoptosis, a ‘knock-in’ allele in *Asah1*, harboring a classical Farber missense mutation (P361R), accurately models most of the characteristics of human disease. This may also indicate a specific role for ASAH1 in murine development. Interestingly, intravenous lentiviral gene transfer expressing human Acid ceramidase in *Asah1*^*P361R/P361R*^ reduced the symptoms, though the mice still succumbed to disease. Conditional *Asah1* floxed mouse have also been generated, which will be helpful in testing cell specificity and efficacy of various therapies (Eliyahu et al., 2012; Alayoubi et al., 2013).
2. Metachromatic leukodystrophy (MLD, OMIM #250100) is due to mutations in *ARSA* (Arylsulfatase A), necessary for the metabolism of sulfatide, causing hypotonia, mental deterioration and cognitive regression. The accumulation of cerebroside sulfate causes demyelination of the frontal and parietal periventricular and central zones (Eichler et al., 2009). Currently, no effective treatment is available to reverse the deterioration and loss of function that MLD causes. An *Arsa*^(–/–)^ mouse was generated which displays a milder neuropathological phenotype than human cases. The model has been useful in showing that attempts to rescue the mild phenotype via various therapies including ERT, HSCT and GT were successful in ameliorating the pathology (Hess et al., 1996). The lack of a complete recapitulation of the clinical and pathological MLD phenotype led to the generation of the *Arsa*^(–/–)^ mice with the addition of neural cells overexpressing the sulfatide synthesizing enzymes, including UDP-galactose: ceramide galactosyltransferase (CGT) and cerebroside sulfotransferase (CST). These *CGT/Arsa^(–/–)^* and *CST/Arsa^(–/–)^* mice had increased sulfatide storage in myelin-forming cells, resulting in axonal degeneration leading to the development of neurological symptoms similar to MLD (Patil and Maegawa, 2013).
3. Krabbe disease (OMIM #245200) is caused by a deficiency of *GALC* (Galactosylceramidase), which leads to the accumulation of psychosine (galactosylsphingosine), a metabolite of galactosylceramide (Li et al., 2019). Clinical symptoms often present in the 1st year of life including irritability, spasticity and developmental delay. Underlying pathology includes widespread demyelination and neurodegeneration (Li et al., 2019). HSCT appears to be of some benefit in cases of later onset or in infantile patients who have been diagnosed before symptoms begin (Wright et al., 2017). Several *Galc* mutant mice have been reported, including two spontaneous mutants *twitcher* (Duchen et al., 1980) and *twi-5J* (Potter et al., 2013), humanized *GALC* transgenic (Gasperi et al., 2004), *GALC-Gly270Asp* (Matthes et al., 2015), *Galc-His168Cys* knock-in mice (Luzi et al., 2001) and Saposin A knockout mice (Matsuda et al., 2001). The *twitcher* mouse (*Galc*^*W339X/W339X*^) is an authentic murine model of Krabbe disease, presenting a near identical neurological phenotype to the human disease (Kobayashi et al., 1980). Due to the highly authentic nature of the *twitcher* model of KD, an incredible number of therapeutic strategies have been tested on *twitcher* mice. These include HSCT (Yeager et al., 1984), neural and mesenchymal stem cell transplantation (Taylor and Snyder, 1997; Strazza et al., 2009; Neri et al., 2011; Ripoll et al., 2011), SRT (LeVine et al., 2000), anti-oxidant therapy (Pannuzzo et al., 2010), PCT (Berardi et al., 2014), ERT (Lee et al., 2005) and GT alone (Shen et al., 2001; Lin et al., 2005; Lee et al., 2007; Rafi et al., 2012, 2015a; Pan et al., 2019). Although viral gene therapy should theoretically be curative, the pre-clinical efficacy *in vivo* has had major limitations. Viral-mediated GT trials in the *twitcher* tends to only modestly improve survival, typically by 1.5-2 fold. Therefore, multiple approaches have been made to improve delivery and transducing ability. These include diffuse and multiple injection points by employing multiple intracerebral points of injection or a combination of intracerebral, intrathecal and intravenous point. Interestingly a recent paper had far better success in the *twitcher* mouse by using much higher doses of AAV9 (Marshall et al., 2018). Additionally, due to the partial effect among different modalities of therapies, a number of groups have approached the concept of combining different forms of therapy. The effect of combining different forms of therapies has had profound improvements of survival of both the *twitcher* and canine models of KD. In particular, a robust synergistic effect of HSCT and viral-directed GT has been noted and replicated by multiple labs (Reddy et al., 2011; Rafi et al., 2015a; Ricca et al., 2015). Furthermore, triple combined therapy of HSCT, GT and SRT with L-cycloserine resulted in an unprecedented increase in lifespan with improved motor function, persistent GALC expression, nearly normal psychosine levels, and decreased neuroinflammation in the *twitcher* (Hawkins-Salsbury et al., 2015), suggesting that simultaneous-treatment of multiple pathogenic aspects of KD may be necessary for synergistic increases in therapeutic efficacy of KD patients. The *twi-5J (Galc ^*E130K/E130K*^)* mice manifest a more severe phenotype with shorter lifespan than the *twitcher* (Potter et al., 2013), indicating the possible existence of a toxic gain-of function mutation from misfolded GALC protein.
4. Gaucher Disease (OMIM #230900) is caused by mutations of *GBA* (Acid β-glucosidase), which leads to the accumulation of glucosylceramide and glucosylsphingosine. Gaucher disease is characterized clinically as types 1-3, with types 2 and 3 having primary CNS disease. Infantile Gaucher disease (type 2) often presents with signs of bulbar and pyramidal neuronal atrophy, as well as cognitive impairment and progressive neurological deterioration and is usually fatal by age 2 (Pastores and Hughes, 2018). Treatment for types 1 and 3 include SRT, and ERT including miglustat (Zavesca) and eligustat tartrate (Cerdelga). PCT with ambroxol, a drug that breaks up phlegm of respiratory diseases, has been evaluated for pre-clinical testing for Gaucher disease (Bendikov-Bar et al., 2013). Unfortunately, there is no effective treatment for the neurologic damage of GD types 2 and 3. HSCT can reverse the non-neurological effects of the disease, but the procedure carries a high risk and is rarely performed in individuals with Gaucher disease (Riboldi and Fonzo, 2019). Interestingly, patients with type 1 are at increased risk for Parkinson’s disease and Lewy Body Dementia. It has been suggested that accumulated glucosylceramide may directly influence amyloid formation of α-synuclein by stabilizing soluble oligomeric intermediates in the lysosome of dopaminergic neurons of *GBA* mutant, that is one of hallmarks of Parkinson’s disease (Mazzulli et al., 2011). Numerous murine models have been created, including *Gba^(–/–)^,Gba^(L444)^,Gba^(pmuts),^* and *Gba*^(lnl/lnl)^. The full KO and *L444P* models both die within 24–48 h of birth due to a compromised endothelial layer (Tybulewicz et al., 1992; Liu et al., 1998). The latter two models were able to circumvent the endothelial lethality barrier by either using cre-driven expression to negate endothelial expression or by using point mutations causing a less severe phenotype (Xu et al., 2003; Enquist et al., 2007). The *lnl* model has been useful for pre-clinical trials with research using PCT, ERT and SRT related therapies being tested on the model. Some of these studies have highlighted limitations also seen in clinical ERT trials, namely the poor distribution of recombinant enzyme penetrating the CNS. Conditional *Gba* floxed mice are also available that have shown cell-specific toxicity in which neuroinflammation is triggered by molecules released from dying neurons, astrocytes and oligodendrocytes (Farfel-Becker et al., 2011; Platt et al., 2018).
5. Type 1 GM1-Gangliosidosis (OMIM #230500) is caused by mutations in *GLB1* (beta-galactosidase-1) leading to the accumulation of GM1 gangliosides and presenting with prompt psychomotor dysfunction and general CNS degeneration within the first 6 months of life. Severe cerebral atrophy leads to death, often within the first 2 years of life (Platt et al., 2018). While there is no effective medical treatment, anti-convulsants may control seizures secondary to GM1 Gangliosidosis. Thus far, two *Glb1*^(–/–)^ mutants have been generated that express disease symptomology consistent with the human disease such as paralysis and premature death (Hahn et al., 1997; Matsuda et al., 1997). These models have been used to study PCT, SRT and HSCT as potential therapeutics which have all shown promise in reversing the neurological phenotype (Kasperzyk et al., 2005; Sano et al., 2005; Elliot-Smith et al., 2008; Takamura et al., 2011).
6. GM2-gangliosidosis, Type I (OMIM #272800; also called as Tay-Sachs Disease) is caused by mutations in *HEXA* (β-hexosaminidase A), which normally degrades GM2 gangliosides, and typically manifests within 6 months of life. Tay-Sachs is characterized by progressive hypotonia, weakness, neurodegeneration and death by 4 years of age. Neuropathology includes less pronounced fissures and enlarged sulci with a great loss of neuronal density in the cerebral cortex along with demyelination of cerebral white matter (Kaback and Desnick, 2011). There is no effective treatment for Tay-Sachs beyond palliative care. Two *HexA*^(–/–)^ murine models have been produced, though none of them seem to recapitulate human disease. Instead, the mouse models are asymptomatic due to a rescue pathway involving Sialidase, not present in humans (Yamanaka et al., 1994; Sango et al., 1995). Recently, a dual KO model *HexA*^(–/–)^ and *Neu3*^(–/–)^ has since been generated that nullifies the bypass pathway via Sialidase (Seyrantepe et al., 2018) and more closely resembles the human disease.
7. GM2-gangliosidosis, Type II (OMIM #268800; also called as Sandhoff disease) is due to mutations in *HEXB* (β-hexosaminidase B), and results in deficiencies of both HexA and HexB enzymatic activities. Sandhoff disease is therefore often indistinguishable from the Tay-Sachs disease phenotype (Platt et al., 2018). Although there is no available specific treatment, supportive treatment such as proper nutrition and hydration, as well as use of anti-convulsants for patients with Sandhoff disease. *HexB*^(–/–)^ mice were developed that recapitulates the pathology seen in human patients. Various approaches including GT and HSCT in the pre-clinical trials in this model showed slow disease progression and prolong lifespan (Sargeant et al., 2011).
8. Niemann-Pick Disease Type A (NPD-A) & B (OMIM #257200) are caused by mutations in *ASM* (Acid sphingomyelinase), which cleaves the phosphorylcholine group from sphingomyelin. NPD-A is categorized as the neuronopathic form of disease, often manifesting in early childhood, while NPD-B does not involve CNS manifestations. Neurologic symptoms of NPD-A include progressive hypotonia, psychomotor developmental regression and relentless neurologic deterioration. There is significant atrophy in the cerebellar and cortical neurons with presentation of foam cells (Vanier, 2013). Two *Asm*^(–/–)^ mouse models have been generated that reproduce human disease symptomology. They have been used to study GT and ERT effects on Niemann-Pick disease (Horinouchi et al., 1995; Otterbach and Stoffel, 1995). These studies have shown limitations in therapeutic techniques such as viral vector-mediated GT as intracerebral injections were insufficient to deliver vectors throughout the CNS, instead requiring intracerebroventricular injections to alleviate motor abnormities.
9. Niemann-Pick disease, Type C1 (OMIM # 257220) and Type C2 (OMIM #607625) are due to mutations in *NPC1* and *NPC2*, respectively. These proteins are involved with the trafficking of cholesterol and lipids within lysosomes and endosomes. Specifically NPC1 is a multipass lysosomal membrane protein that transports sphingosine out of lysosomes, whereas NPC2 is a soluble cholesterol-binding protein. NPC causes a secondary reduction of ASM, thus producing overlapping symptoms with NPD-A and B. Patients with NPC presents with mental degeneration, dementia and dystonia due to neuronal atrophy, particularly present in the Purkinje cells of the cerebellum (Vanier, 2013; Platt et al., 2018). Supportive care is essential and substantially improves the quality of life of NPC patients. Numerous murine models of NPC have been generated including *Npc1*^(–/–)^, as well as multiple missense knockin models (*D1005G, P202A, F203A, I1061T*, and *1554-1004 G* > *A*) and a conditional floxed model. The latter has been used for the cell-specific knockout of *Npc1* in Purkinje cells (Morris et al., 1977; Elrick et al., 2010; Xie et al., 2011; Maue et al., 2012; Praggastis et al., 2015; Gómez-Grau et al., 2017). Depending on the point mutation induced, many of the models fully recapitulate the human disease phenotype including neurodegeneration, shortened lifespan and Purkinje cell atrophy. The *I1061T* mutant, which harbors the most common human NPC1 mutation, was generated to specifically study the effects of proteostatic modulation on disease progression. Various preclinical trials including GT, ERT, and transplant in these models have shown different but promising levels of success in prolonging mutant lifespan, reducing Purkinje cell atrophy and the storage phenotype (Veyron et al., 1996; Soga et al., 2015; Chandler et al., 2017b; Xie et al., 2017; Hughes et al., 2018).

#### Lysosomal Transport Defects

Whereas a majority of LSDs are the result of a defunct catabolic enzyme unable to metabolize its substrate, lysosomal transport defects are the results of mutations to intracellular membrane transporters. The absence of specific transmembrane transporters can lead to substrate being trapped in the lysosome, barred from cellular recycling. The two most common forms of lysosomal transport defects are Cystinosis and Sialic Acid storage disorders (Mancini et al., 2000).

1. Cystinosis (OMIM #219800) is due to mutations in *CTNS* (Cystinosin), which normally allows for the exit of cystine from the lysosome. Cystinosis has primarily nephropathic sequelae including renal tubular Fanconi syndrome (generalized proximal tubular dysfunction) and progressive glomerular failure. Advances in therapy including cystine-depleting agents and renal transplantation have allowed patients to survive longer and revealed some neurologic dysfunction and cerebellar calcification (Nesterova and Gahl, 2017). A *Ctns*^(–/–)^ mouse model was developed that displays some of the secondary symptoms of Cystinosis, though it lacks the primary ailment in renal failure. Nonetheless, the murine model has still been useful in testing potential therapeutics such as HSCT and medicinal treatments such as Cysteamine, the first line of defense drug against cysteine accumulation (Cherqui et al., 2002; Syres et al., 2009; Simpson et al., 2011; Rocca et al., 2015; Gaide Chevronnay et al., 2016).
2. Sialic acid storage disorders (infantile free sialic acid storage disease [ISSD]; OMIM #269920, Salla disease; OMIM #604369) are a spectrum of disorders due to mutations in *SLC17A5* (Sialin), which are autosomal recessive neurodegenerative disorders that present as a severe infantile form (ISSD) or as a slowly progressive adult form (Salla disease). General symptoms include developmental delay, low muscle tone, abnormal movements, and seizures. They are progressive, and symptoms get worse over time. Sialin is a sialic acid lysosomal membrane transport protein (Adams and Gahl, 2013). Since there is no cure, supportive treatments including anti-convulsants and physical therapy are recommended for the patients. Recently developed *Slc17a5*(-/-) mice successfully show early neurobehavioral milestones including hypomyelination and leukoencephalopathy, but exhibit progressive delay of later-stage sensory and motor milestones such as grasping, twitching and locomotion development (Stroobants et al., 2017).

#### Multiple Enzyme Deficiency

Multiple enzyme deficiency LSDs refer to those LSDs where more than one enzyme involved in the catabolism of lipids may be indirectly affected, thus eliciting a pathology associated with multiple LSDs due to a single mutation (Schlotawa et al., 2019).

1. Multiple sulfatase deficiency (MSD, OMIM #272200) is due to a mutation in *SUMF1* (sulfatase modifying factor 1) resulting in a dysfunctional or complete absence of formylglycine-generating enzyme (Fge). Fge is responsible for post-translationally activating sulfatase enzymes that could affect the activity of all 13 sulfatase enzymes. While symptoms of MSD patients can be highly variable, many may display characteristics of the mucopolysaccharidoses, including developmental regression and neurologic deterioration (Schlotawa et al., 2019). There is no cure for MSD. Similarly to MSD patients, *Sumf1*(-/-) mice display early mortality, congenital growth retardation, skeletal abnormalities, and neurological defects including widespread neurodegeneration and neuroinflammation (Settembre et al., 2007). Combined brain and systemic AAV mediated GT in this mouse model resulted in significant improvement in both growth rate and lifespan (Surace et al., 2007; Spampanato et al., 2011).
2. Mucolipidosis (ML), Types II (I-Cell disease, OMIM #252500), III α/β (Pseudo-Hurler polydystrophy, OMIM #252600) and III gamma (OMIM #252605) are caused by mutations in *GNPTAB* (for ML II, ML III α/β) and *GNPTG* (ML III γ). GNPTAB and GNPTG give rise to the enzyme *N-*acetylglucosamine-1-phosphotransferase, a phosphotransferase which normally phosphorylates mannose residues for proper enzymatic trafficking of hydrolases to the lysosome. In addition to widespread musculoskeletal and cardiac phenotypes, patients with ML II have delays in motor milestones and expressive language (Leroy et al., 2019). There is no specific therapy to cure ML II and III. Speech and physical therapies can improve motor and speech delays. The *Gnptab(-/-)* mouse showed impaired growth, retinal degeneration, lesions in secretory epithelial cells of exocrine glands, and elevated levels of serum acid hydrolases. However, this mutant presented with a relatively normal lifespan and did not develop characteristic disease features, such as skeletal and facial abnormalities (Vogel et al., 2009). Another mouse model *Nymphe* (*nym/nym*), which was recovered from an N-ethyl-N-nitrosourea screen, and carries the patient mutation *Y888X*, recapitulates the major features of the human disease including motor dysfunction and psychomotor retardation with progressive neurodegeneration of Purkinje cells. Treatment with 2-hydroxypropyl-β-cyclodextrin delayed Purkinje cell loss in a NPC model, but had no effect on Purkinje cell loss in the *Nymphe* mouse. This finding suggested that the loss of *Npc2* (Niemann-Pick, Type C2) expression in the *Nymphe* mouse brain is not a primary molecular mechanism causing Purkinje cell degeneration (Paton et al., 2014).
3. Mucolipidosis, Type IV (MLIV) is caused by mutations in *MCOLN1* (Mucolipin 1), an endo-lysosomal pH sensitive channel that facilitates diffusion of monovalent and divalent ions (Dong et al., 2008) and facilitates lysosomal and autophagic regulation via its interaction with TFEB and mTOR signaling (Zhang et al., 2016). Clinical findings often include developmental delay, gross psychomotor impairments and failure to reach developmental milestones. There is no specific treatment to this disorder. Neurological impairments are attributed to developmental dysregulation of the corpus callosum in addition to atrophy and axonal spheroids in the cerebellum (Schiffmann et al., 2014). An authentic murine model of MLIV, *Mcoln1*^(–/–)^, has been generated and closely resembles the pathology seen in human patients including hind limb paralysis and reduced lifespan (Venugopal et al., 2007). The model has been used to study the effects of HSCT and drug administration to improve the neuropathology associated with the disease with promising results (Walker and Montell, 2016).

#### Glycogen Storage Disease

Glycogen storage diseases are caused by impaired glycogen degradation or synthesis. Of the 13 known glycogen storage diseases, only one fits the criteria of classification as an LSD, namely Glycogen storage disease (GSD) II. GSD II is typically referred as Pompe disease, though the variant GSD-Type IIb (Danon disease) also exists and shares a similar phenotype. There is debate as to whether Danon disease should be classified as a glycogen storage disease as the mutation occurs in a lysosomal membrane protein and glycogen accumulation is not always present (Nishino et al., 2000).

1. Pompe disease (GSD II, OMIM #232300) is caused by mutations in *GAA* (Acid α-Glucosidase), which encodes an enzyme that degrades the α-1,4 and α-1,6 linkages required for the degradation of glycogen. The infantile-onset form of disease presents with hypotonia, feeding difficulties and is fatal due to cardiorespiratory failure. The effects on the CNS are still obscure due to the early age of death, however, research has shown a delay in reaching myelination milestones in patients (Chien et al., 2006). ERT of Myozyme (alglucosidase alfa) clearly prolongs overall survival for infantile-onset Pompe disease. Early diagnosis and early treatment leads to much better outcomes (Capelle et al., 2018). ERT with Lumizyme (alglucosidase alfa) is approved for patients with late-onset Pompe disease without evidence of cardiac hypertrophy, which improves survival and ambulation maintained over time. Lumizyme and Myozyme have the same generic ingredient (alglucosidase alfa; recombinant human GAA) and manufacturer (Genzyme Co.), but have the difference in the manufacturing process (Schoser et al., 2017). Two authentic murine *Gaa(-/-)* models have been established, and both of them have analogous pathology to the human disease with hallmarks such as muscle weakening and gait abnormalities (Bijvoet et al., 1998; Raben et al., 1998). The models have proven useful in pre-clinical trials with a variety of treatments including gene therapy, BMT, PCT, ERT and glycogen accumulation suppression (Raben et al., 2001, 2002, 2010; Rucker et al., 2004; Xu et al., 2004; Zhu et al., 2005; Mori et al., 2008; Khanna et al., 2014; Gatto et al., 2017; Puzzo et al., 2017).
2. Danon disease (GSD IIb, OMIM #300257) is an X-linked disease caused by mutations in *LAMP2* and has overlapping symptoms with Pompe disease, including diffuse hypotonia and hypertrophic cardiomyopathy. However, unlike Pompe disease, most male patients with Danon disease have mild intellectual disability (Cenacchi et al., 2019). There is no approved treatment. A mouse model of *Lamp2* knockouts exist and have inflammation, motor deficits and impaired learning (Rothaug et al., 2015). AAV-mediated GT increased survival rate of this mutant (Tanaka et al., 2000; Adler et al., 2019).

#### Neuronal Ceroid-Lipofuscinoses

The final category of inherited, neurodegenerative, LSDs include the neuronal ceroid-lipofuscinoses (NCLs). They are generally characterized clinically by neurologic deterioration, seizures and early death. While the NCL phenotypes resemble each other, there exists broad genetic heterogeneity and multiple mechanisms of pathogenesis. To date, thirteen genes have been identified to cause NCLs including *PPT1, TPP1, CLN3, CLN5, CLN6, MFSD8, CLN8, CTSD, DNAJC5, CTSF, ATP13A2, GRN*, and *KCTD7* (Mole and Williams, 2013). While various therapeutic strategies are being explored for NCLs, there is only one clinically approved drug, cerliponase alfa, that effectively attenuates the progression of a specific form of NCLs (CLN2) (Kohlschütter et al., 2019).

1. CLN1 (OMIM #256730; infantile NCL, Santavuori-Haltia) is caused by mutations in *PPT1*, which encodes Palmitoyl-protein thioesterase-1, responsible for the catabolism of thioester-linked fatty acyl groups from cysteine residues. CLN1 presents with infantile developmental delays, myoclonic jerks and seizures. The symptoms are due to neuronal atrophy, particularly in the cortex and cerebellum (Mole et al., 2005). Three murine models have been established including a full null *Ppt1*^(–/–)^ and two common missense mutations *Ppt1*^(R151X)^ and *Ppt1*^(C451T)^ (Gupta et al., 2001). All of them have been shown to recapitulate many of the pathological features observed in CLN1 patients, such as accumulation of autofluorescent granular osmiophilic deposits in neural and visceral tissues, rapidly progressing neurodegeneration in the brain, motor abnormalities, seizures and premature death. These models have been used in the development of therapies such as GT, dietary supplements, pharmacological and ERT. The combination of these therapies has additionally been shown to prolong the diseased animals’ lifespan. *Ppt1*^(R151X)^ mutant mice treated with the read-through drug ataluren (PTC124) have been shown to increase CLN1 enzyme activity (Miller et al., 2015).
2. CLN2 (OMIM #204500, late-infantile NCL, Jansky–Bielschowsky) is caused by mutations in *TPP1*, which encodes tripeptidyl peptidase 1, involved in the cleavage of N-terminal tripeptides. Patients with CLN2 develop symptoms between the age of 2 to 4 years, including ataxia, seizures and mental deterioration. The formation of curvilinear body, that is an intermingled twisted microtubular substructure, is a hallmark of the disease and is often accompanied by cortical thinning (Mole and Williams, 2013). Intrathecal ERT delivery of cerliponase alfa (a recombinant human proenzyme of TPP1) is clinically approved for affected children (Kohlschütter et al., 2019). The *Cln2*^(–/–)^ mouse recapitulated the clinical course of CLN2 with axonal degeneration, Purkinje cell atrophy and a reduced lifespan. The model has been used to study GT, ERT and anti-apoptosis manipulation all with varying degrees of success (Kohlschütter and Schulz, 2016).
3. CLN3 (OMIM #204200, juvenile NCL, Spielmeyer-Vogt) is caused by mutations in the *CLN3* gene, which encodes a lysosomal transmembrane protein. Symptoms are progressive, and include visual impairments between 2 to 4 years of age and epilepsy with generalized tonic-clonic seizures after 9 years of age, as well as ataxia and motor dysfunction (Mole et al., 2005). Three full KO models of *Cln3* have thus far been established all displaying hallmarks of CLN3 such as progressive neurodegeneration (Mitchison et al., 1999; Cotman et al., 2002; Eliason et al., 2007). To date there has been research using these models in alleviating CLN3 pathology with administration of various anti-inflammatory and neuroprotective compounds such as ibuprofen and lamotrigine to modulate intracellular inflammatory conditions (Mirza et al., 2019; Tarczyluk-Wells et al., 2019).
4. CLN6 (OMIM #601780, adult NCL) is caused by mutations in *CLN6*, a transmembrane protein of the endoplasmic reticulum. Patients with CLN6 generally begin to exhibit symptoms before the age of 40 (mean age 28). Clinical manifestations of disease first present as myoclonus epilepsy, followed by ataxia and dementia due to cerebral and cerebellar atrophy (Berkovic et al., 2019). A neuronal ceroid lipofuscinosis (*Nclf*) murine model was established by a one base pair insertion in the orthologous mouse *Cln6* gene resulting in a frame shift defect that recapitulates many aspects of the disease such as Wallerian degeneration and paralysis (Bronson et al., 1998). This model has been used to research the effects of GT and diet intervention on disease progression (Kohlschütter et al., 2019).

## Discussion

### Limitations of Using Mouse Models to Study Human LSDs

Mouse models have been used for biomedical research since the beginning of the 20th century (Ericsson et al., 2013). These models have been used widely for a number of reasons including their relative low cost, high level of reproducibility, and short lifespans. Furthermore, the similarity of the mouse and human genome have facilitated the study of human disease in murine models. Early advances in modern mouse models began with the development of transgenic mice and knockout mice, which progressed to the advance of conditional mutagenesis (Orban et al., 1992), inducible mutagenesis (Doetschman and Azhar, 2012) and fluorescent reporters (Ikawa et al., 1995). Consequentially, the use of mouse models has drastically outpaced the use of rats and other mammals for modeling of human disease (Ericsson et al., 2013). Furthermore, modern techniques and services have rapidly increased the speed and decreased the cost of producing mouse models of interest.

It is crucial to have highly predictive animal models as pre-clinical test tools for the development of LSD therapies, where small patient sample sizes and variable ages of presentation make it difficult to standardize endpoint evaluation in clinical tests. While many mouse models of LSDs accurately reflect the human symptomatology of the disease they model, there are a number of examples in which generated models fall short. For example, *Arsa*^(–/–)^ MLD mice show mild neurological symptoms that are only observed by the end of a normal lifespan, and fail to show sulfatide accumulation and demyelination (Patil and Maegawa, 2013). These differences may be explained by the fundamental differences of mice and human development, particularly in regards to neurodevelopment (Lin et al., 2014). Despite the homology of genes between different species, it is obvious that there are many variables that are not well replicated by simply introducing the same genetic mutation responsible for a human disease into a mouse model. Attempts to generate a more ‘authentic’ human model may consequentially produce a mouse model similar to the human disease, but nonetheless artificial. For example, *Arsa*^(–/–)^ mice with neural cells overexpressing the sulfatide synthesizing enzyme CGT or CST have more similar pathology to human MLD patients (Patil and Maegawa, 2013). Furthermore, the artificial nature of ‘knockout’ systems sometimes trigger compensatory mechanisms not seen in human disease (El-Brolosy and Stainier, 2017).

Since naturally occurring spontaneous mutant mice are rare and, the vast majority of LSD mice are knockout or knock-in animals. While these models have provided us with significant advances in our understanding of pathogenesis and therapy, these artificial disease models have limitations. Firstly, complete knockouts have limited relevance to the human phenotype, as most patients expressing a low level of functioning or malfunctioning protein. These complete null genotypes occasionally make it hard to reintroduce target proteins, such as gene− or enzyme−replacement therapy, as the novel protein could trigger a neutralizing immune response. This may result in confusion in predicting the efficacy of such treatments in patients. Moreover, not all LSDs are associated with loss−of−function mutations, several are associated with toxic gain−of−function mutations which are often overlooked.

Particularly disconcerting is the high number of ‘successful’ pre-clinical trials that are not successful when replicated in human clinical trials. An analysis of the reported causes of drug candidate attrition during 2013–2015 revealed that past failures in phase II clinical trials were primarily due to insufficient efficacy (48%) and safety (25%). Similarly, insufficient efficacy was the primary reason for failure in phase III (55%), followed by safety (14%). When examining these failures by therapeutic areas, 13% and 17% of the failures were in metabolic and CNS diseases, respectively (Harrison, 2016). There are a number of potential attributable factors to this problem. One explanation for this gap in translation is the lack of robustness in the conduction of the pre-clinical trials. The efficiency of the new treatment under the different conditions indicates the treatment’s robustness (Carlson and Doyle, 2002; Xu et al., 2016). In many cases, pre-clinical trials are not being designed to influence human clinical trials and lack crucial features, such as statistical power, blinding, and appropriately sized control groups (Freise et al., 2011). In addition, design of pre-clinical animal experiments do not reflect all aspects of the challenge originated from the varied age and complex genetic makeup of patients (Lowenstein and Castro, 2009). For example, fixed genetic strains of inbred mice minimize the complexity of some disease phenotypes. Genetic backgrounds of mice have been shown to directly influence disease pathology and survival in models of LSDs (Tominaga et al., 2004), and can therefore present a confounding variable if not properly taken into account. If it is shown that a particular treatment works not only in certain mouse strain but also in additional mouse genetic backgrounds, that will increase the robustness of the treatment. Furthermore, the small size of mice, and in particular mice brains, compared to humans, potentially allows for easier treatment of mice. Alternative approaches include more rigorous testing of pre-clinical trials and the use of large animal models (Techiryan et al., 2018). Additional technologies such as induced pluripotent stem cells and organoids are particularly appealing as using cells from humans patients can more accurately model certain aspects of disease.

### Perspectives

The past three decades have given rise to significant progress in the treatment of LSDs, and thus different therapeutic options are now available and are being actively pursued. Unfortunately, many LSDs remain untreatable and most of the therapeutics offered have important limitations related to a number of issues including bioavailability, toxicity and limited efficacy. Nonetheless, the success of a number of innovative and promising studies is suggestive that many of these LSDs will have novel treatments in the coming years. The possibility of combining different approaches in order to maximize therapeutic efficacy, as well as the ability to personalize treatment options for each individual patient are exciting realities. The use of mouse models continues to be an invaluable tool in the advancement of therapeutic interventions for LSDs.

## Author Contributions

JF, NW, and DS collected all information of LSD animal models and JF, NW, MF, and DS wrote the manuscript.

## Conflict of Interest

The authors declare that the research was conducted in the absence of any commercial or financial relationships that could be construed as a potential conflict of interest.
